# Expanding human variation at *PLOS Genetics*

**DOI:** 10.1371/journal.pgen.1010070

**Published:** 2022-02-14

**Authors:** Gregory S. Barsh, Gregory P. Copenhaver, Hua Tang, Scott M. Williams

**Affiliations:** 1 HudsonAlpha Institute for Biotechnology, Huntsville, Alabama, United States of America; 2 Department of Genetics, Stanford University School of Medicine, Stanford, California, United States of America; 3 Department of Biology and the Integrative Program for Biological and Genome Sciences, University of North Carolina at Chapel Hill, Chapel Hill, North Carolina, United States of America; 4 Departments of Population and Quantitative Health Sciences, Institute of Computational Biology, Case Western Reserve University, Cleveland, Ohio, United States of America

The “experiments of nature” that underlie genetics engage all organisms equally: from microbes and slime molds to plants and vertebrates, the inextricable connection between genotype and phenotype lies at the core of our community. That community is remarkably diverse, spanning an array of not only organisms but also approaches and questions; indeed, diversity is one of the main reasons we enjoy contributing to the journal.

Of course, one leaf on the tree of life carries a special kind of weight, and while *PLOS Genetics* receives, considers, and publishes submissions on human genetics, we have come to realize that we can and should do a better job of letting our authors, readers, and reviewers know that. As a result, we are making a change to emphasize the variety of ways we embrace work on variation, including an emphasis on the importance of human genetics. To that end, we have recruited several new associate editors with relevant expertise, and we are changing the name of our Natural Variation section to ***Human Genetic Variation and Disease***.

Natural Variation was one of the original sections when the journal was founded 16 years ago. Led by Greg Gibson, it balanced phenotypic (both organismal and molecular) and genotypic (common, rare, structural) variation across the organismal spectrum. The section was also a home for human genetics, including studies of mendelian and common disease, normal variation, demography, and genetic architecture. The two of us who currently lead the section work primarily with humans, and while we appreciate and enjoy submissions that focus on all organisms, it has become clear that it makes sense to codify and further strengthen the ability of the journal to serve that portion of our community interested in the “leaf with a special kind of weight”.

**Fig 1:**
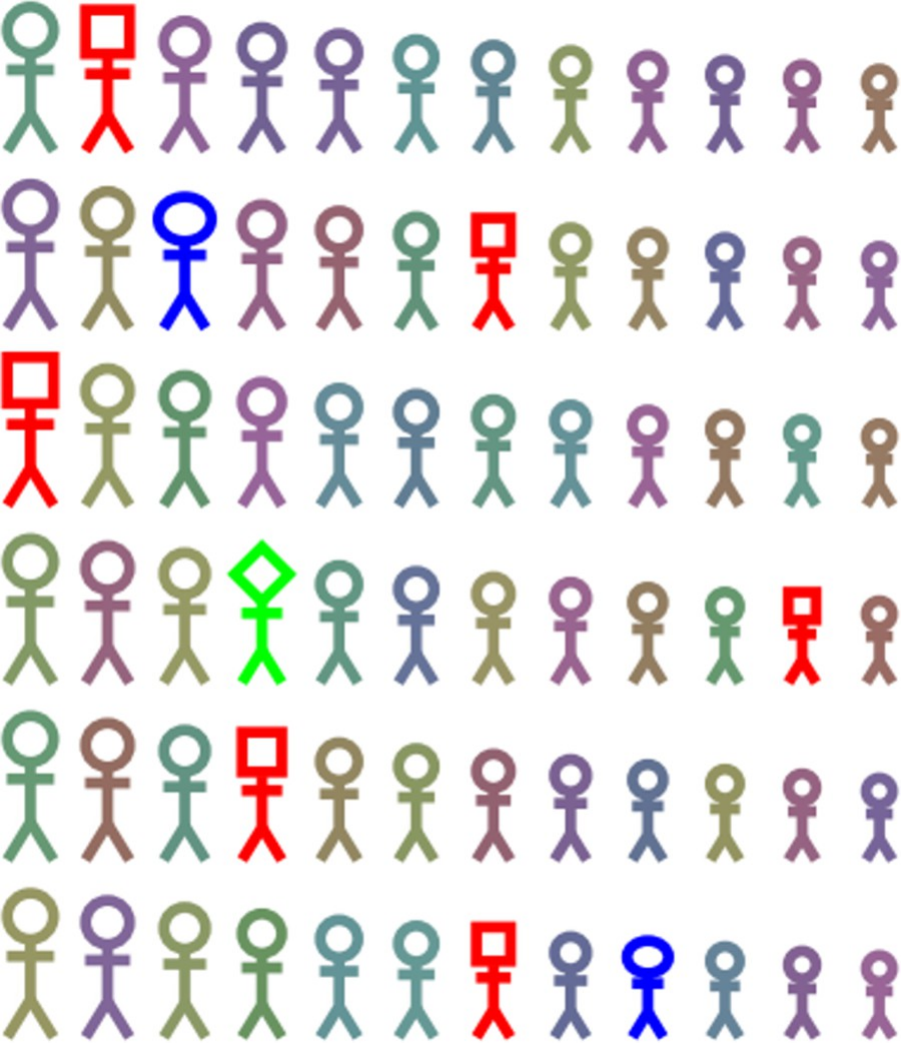
Common variation in quantitative traits such as height, represented by size, and blood pressure, represented by different colors, present a diverse background of traits and disease susceptibility, upon which rare phenotypes are observed, represented by different shapes and colors. The Human Genetic Variation and Disease section will support manuscripts on any (or all) of these aspects of human genetics.

Therefore, along with a change in name, we have recruited several new Associate Editors to the board, including Karen Avraham (diseases of hearing), Neil Hanchard (complex diseases of childhood), Sudha Iyengar (genomics of disorders with complex inheritance), Anne O’Donnell-Luria (genomics of rare variation), and Melissa Wasserstein (medical genetics and inborn errors of metabolism) [1]. These new board members enrich the breadth and depth of the editorial board, particularly when it comes to human disease. Historically, human genetics has been kindled by, in the words of Archibald Garrod [2], “the lessons of rare maladies”, but in a world of exponential accumulation of genome sequence and, critically, phenotype data, common diseases have become increasingly tractable (Fig 1). *PLOS Genetics* anticipates and expects to see and feature manuscripts on both rare and common disease, as exemplified by the interests of our new associate editors.

What about the rest of the tree of life? We remain committed to publishing work on genetic diversity across all 5 kingdoms. We expect that some submissions may fit well with existing sections such as Epigenetics, Evolution, Methods, Plant Genetics, and Prokaryotic Genetics, as well as the “general genetics” section. More broadly, we view the 7 sections at *PLOS Genetics* as an overlapping Venn diagram among which there is frequent and vibrant interchange, another reason the two of us who lead the “general genetics” section enjoy contributing to the journal. Indeed, Garrod quoted another famous physician, William Harvey, whose words in 1657 were inspired by human disease but apply equally well to all leaves of the tree, “Nature is nowhere more accustomed more openly to display her secret mysteries than in cases where she shows traces of her workings apart from the beaten path” [2].
